# SARS-CoV-2 in silico binding affinity to human leukocyte antigen (HLA) Class II molecules predicts vaccine effectiveness across variants of concern (VOC)

**DOI:** 10.1038/s41598-022-11956-5

**Published:** 2022-05-16

**Authors:** Spyros A. Charonis, Lisa M. James, Apostolos P. Georgopoulos

**Affiliations:** 1 The HLA SARS-CoV-2 Research Group, Department of Veterans Affairs Health Care System, Brain Sciences Center (11B), Minneapolis VAHCS, One Veterans Drive, Minneapolis, MN 55417 USA; 2 Department of Neuroscience, University of Minnesota Medical School, Minneapolis, MN 55455 USA

**Keywords:** Immunology, Diseases, Medical research, Mathematics and computing

## Abstract

There is widespread concern about the clinical effectiveness of current vaccines in preventing Covid-19 caused by SARS-CoV-2 Variants of Concern (Williams in Lancet Respir Med 29:333–335, 2021; Hayawi in Vaccines 9:1305, 2021), including those identified at present (Alpha, Beta, Gamma, Delta, Omicron) and possibly new ones arising in the future. It would be valuable to be able to predict vaccine effectiveness for any variant. Here we offer such an estimate of predicted vaccine effectiveness for any SARS-CoV-2 variant based on the amount of overlap of in silico high binding affinity of the variant and Wildtype spike glycoproteins to a pool of frequent Human Leukocyte Antigen Class II molecules which are necessary for initiating antibody production (Blum et al. in Annu Rev Immunol 31:443–473, 2013). The predictive model was strong (r = 0.910) and statistically significant (*P* = 0.013).

## Introduction

In the short time since being designated VOC, the SARS-CoV-2 Omicron variant (B.1.1.529.1) has rapidly and exponentially spread across the globe^1–3^. Relative to previous VOC including Alpha (B.1.1.7), Beta (B.1.351), Gamma (P.1), and Delta (B.1.617.2), Omicron is characterized by a large number of mutations, many involving the spike protein, that appear to increase transmissibility and facilitate immune evasion rendering preventive vaccines, previously acquired natural immunity, and therapeutic monoclonal antibodies significantly less effective than with previous variants^2,4–13^. These concerning effects have consequently prompted calls for updated, more effective vaccines to bring the pandemic under control^14^.

An important consideration with regard to vaccine effectiveness centers around an individual’s ability to mount an immune response that results in antibody production, a process that depends on an individual’s HLA Class II composition. Each individual possesses 6 classical HLA Class II alleles (DRB1, DQB1, DPB1) that encode the HLA Class II cell-surface receptor molecules; these proteins present bound peptides derived from viral antigens to CD4 + T cells to initiate antibody production^3^. However, successful engagement of CD4 + T cells hinges on a good, high binding affinity match between the HLA Class II molecule and the viral protein. In the absence of a match, antibody production and, consequently, an individual’s vaccine response, is impaired.

The relevance of HLA class II to vaccine effectiveness has been documented for several viruses including influenza^15^, HIV^16^, measles^17^, mumps^18^, and hepatitis B and C^19,20^. HLA variability is presumably similarly related to SARS-CoV-2 vaccine effectiveness. To our knowledge, effects of HLA alleles on SARS-CoV-2 vaccine effectiveness have not been investigated in clinical studies; however, we have documented variability of in silico binding affinities of SARS-CoV-2 spike glycoprotein epitopes to common HLA Class II alleles^21^ and identified candidate epitopes shared across SARS-CoV-2 variants that bind with high affinity to Class II HLA alleles which could inform successful multivalent SARS-CoV-2 vaccine design^22^. With regard to effectiveness of the current SARS-CoV-2 vaccines, an additional consideration involves the degree of similarity of HLA binding between the Wildtype strain, against which vaccines were developed, and VOC. Research has demonstrated decreased vaccine effectiveness with successive VOC with the highest efficacy found for Alpha, followed by Beta, Gamma, and then Delta^2^. It is unclear to what extent decreased vaccine effectiveness may be associated with alterations in HLA binding as the virus evolves. Here we determined the extent of overlapping high-affinity HLA binding for each VOC with Wildtype SARS-CoV-2 and evaluated the correspondence between that overlap and reported vaccine effectiveness against VOC in order to predict vaccine effectiveness against Omicron.

## Materials and methods

### SARS-CoV-2 variants

The SARS-CoV-2 variants and some of their sequence properties are summarized in Table 1. The amino acid sequences of all viral spike glycoprotein variants (Alpha, Beta, Gamma, Delta, Omicron) were retrieved from the NCBI SARS-CoV-2 data hub (www.ncbi.nlm.nigh.gov/sars-cov-2). The sequence of the Wildtype spike glycoprotein was retrieved from the UniProtKB database^23^.

**Table 1:** SARS-CoV-2 spike glycoprotein variants.

Variant/Location	Nomenclature	Length of viral protein	N of 15-mers	N of 18-mers	N of 22-mers
Wildtype	SARS-CoV-2	1273	1258	1255	1251
UK (Alpha)	B.1.1.7	1271	1256	1253	1249
South Africa (Beta)	B.1.351	1273	1258	1255	1251
Brazil (Gamma)	P.1	1273	1258	1255	1251
India (Delta)	B.1.167.2	1271	1256	1253	1249
South Africa (Omicron)	B.1.1.529	1270	1255	1242	1248

### HLA alleles

For this study, we selected the more frequent alleles of classical HLA Class II genes (DPB1, DQB1, and DRB1), namely all alleles with frequencies ≥ 0.01, an arbitrary but reasonable threshold. For that purpose, we obtained an Estimation of Global Allele Frequencies by querying the relevant website (http://www.allelefrequencies.net). The alleles with frequencies ≥ 0.01 that we used are listed in Table 2. They comprised 21, 15 and 30 alleles of DPB1, DQB1 and DRB1 genes, respectively.

**Table 2:** HLA class II alleles used (frequency ≥ 0.01).

Index	Allele	Gene	Frequency
1	DPB1*01:01	DBP1	0.02473
2	DPB1*02:01	DBP1	0.16181
3	DPB1*02:02	DBP1	0.02725
4	DPB1*03:01	DBP1	0.01356
5	DPB1*04:01	DBP1	0.23022
6	DPB1*04:02	DBP1	0.05857
7	DPB1*05:01	DBP1	0.02403
8	DPB1*06:01	DBP1	0.01524
9	DPB1*09:01	DBP1	0.07696
10	DPB1*10:01	DBP1	0.01642
11	DPB1*101:0	DBP1	0.02327
12	DPB1*107:0	DBP1	0.01149
13	DPB1*11:01	DBP1	0.05043
14	DPB1*13:01	DBP1	0.02144
15	DPB1*14:01	DBP1	0.01001
16	DPB1*17:01	DBP1	0.11305
17	DPB1*18:01	DBP1	0.16296
18	DPB1*21:01	DBP1	0.01295
19	DPB1*22:01	DBP1	0.15451
20	DPB1*28:01	DBP1	0.06760
21	DPB1*77:01	DBP1	0.01827
22	DQB1*02:01	DQB1	0.05185
23	DQB1*02:02	DQB1	0.09970
24	DQB1*03:01	DQB1	0.01745
25	DQB1*03:02	DQB1	0.24528
26	DQB1*03:03	DQB1	0.05454
27	DQB1*04:01	DQB1	0.04042
28	DQB1*04:02	DQB1	0.02836
29	DQB1*05:01	DQB1	0.05585
30	DQB1*05:02	DQB1	0.02224
31	DQB1*05:03	DQB1	0.02484
32	DQB1*06:01	DQB1	0.01448
33	DQB1*06:02	DQB1	0.13308
34	DQB1*06:03	DQB1	0.04115
35	DQB1*06:04	DQB1	0.01172
36	DQB1*06:09	DQB1	0.01460
37	DRB1*01:01	DRB1	0.04195
38	DRB1*01:02	DRB1	0.02500
39	DRB1*03:01	DRB1	0.02248
40	DRB1*04:01	DRB1	0.07162
41	DRB1*04:02	DRB1	0.04415
42	DRB1*04:03	DRB1	0.07516
43	DRB1*04:04	DRB1	0.01597
44	DRB1*04:05	DRB1	0.02097
45	DRB1*04:07	DRB1	0.08850
46	DRB1*04:11	DRB1	0.04420
47	DRB1*07:01	DRB1	0.10029
48	DRB1*08:01	DRB1	0.05065
49	DRB1*08:02	DRB1	0.08724
50	DRB1*08:03	DRB1	0.06829
51	DRB1*09:01	DRB1	0.01421
52	DRB1*10:01	DRB1	0.01807
53	DRB1*11:01	DRB1	0.02435
54	DRB1*11:04	DRB1	0.04195
55	DRB1*12:01	DRB1	0.01429
56	DRB1*12:02	DRB1	0.03210
57	DRB1*13:01	DRB1	0.03344
58	DRB1*13:02	DRB1	0.02426
59	DRB1*13:03	DRB1	0.01362
60	DRB1*14:01	DRB1	0.01629
61	DRB1*14:02	DRB1	0.04806
62	DRB1*14:54	DRB1	0.02014
63	DRB1*15:01	DRB1	0.05119
64	DRB1*15:02	DRB1	0.01335
65	DRB1*16:01	DRB1	0.03554
66	DRB1*16:02	DRB1	0.04270

### Partitioning the SARS-CoV-2 spike glycoprotein variants

**Figure 1:**
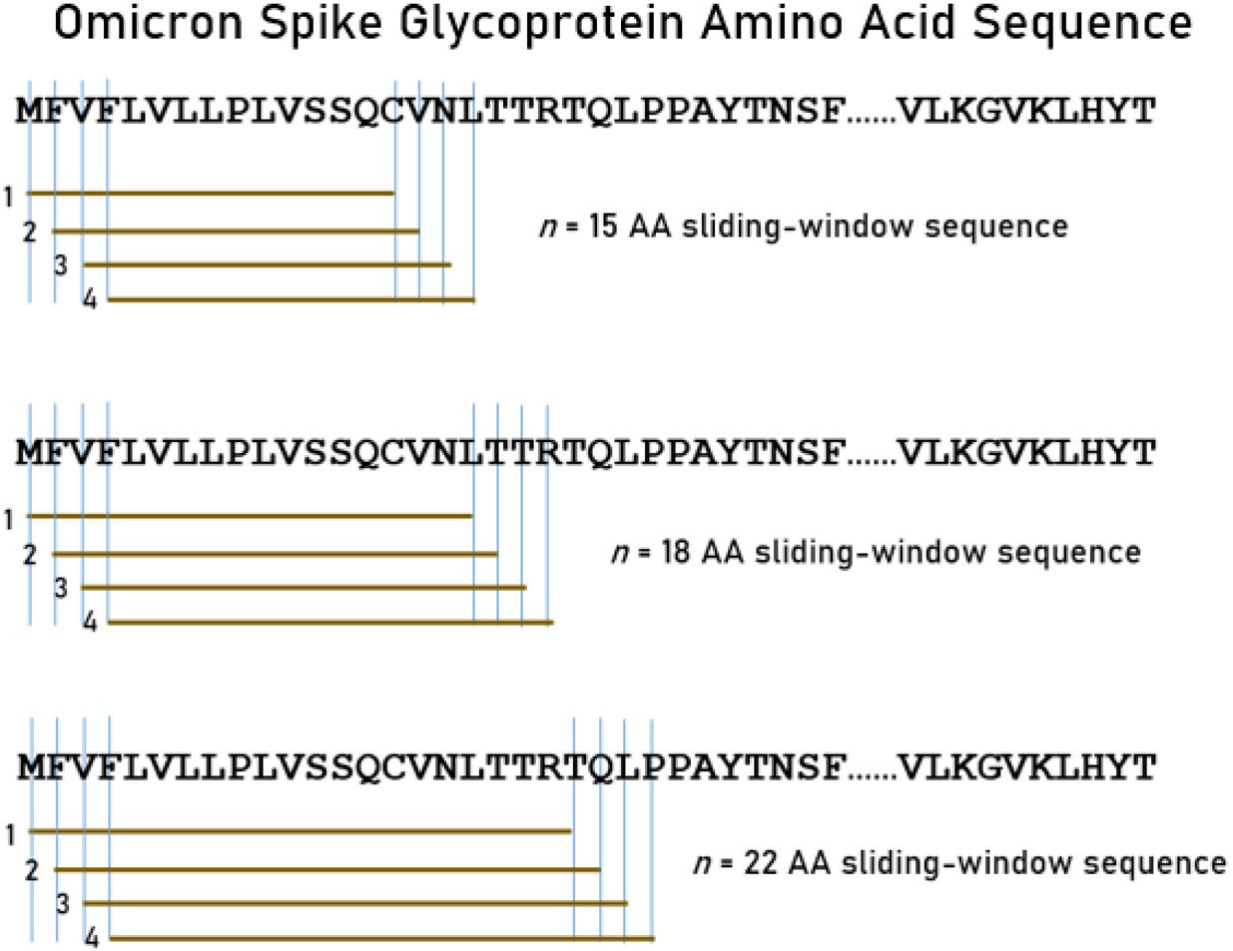
A sample of the sliding window approach^21^ for the Omicron spike glycoprotein.

The complete spike glycoprotein sequence was analyzed for all variants; the full sequences of all variants analyzed are given in the Appendix. Each viral sequence was queried for binding affinity against 66 common HLA Class II alleles (Table 2). A sliding epitope window approach^21^ was used to partition the sequence of the spike glycoprotein for each variant. Partitioning was done in a manner to obtain all possible consecutive linear 15-, 18- and 22-mers (e.g. for 15-mers residues 1–15, 2–16, …, *n*−15 where *n* = sequence length) that cover the entire sequence length (Fig. 1). These peptide lengths are in the range of suitable lengths for binding with HLA Class II molecules^3^. Hence, each linear n-mer was treated as a putative epitope whose binding affinity was to be predicted. The partitioning was implemented using a Python script (version 3.8).

### Determination of binding affinity

All *n*-mers were queried in the Immune Epitope Database (www.iedb.org) in order to determine their binding affinities to the set of 66 HLA Class II molecules (Table 2). Binding affinity predictions were obtained using the NetMHCIIpan method^24^ which covered all 66 alleles used. For each *n*-mer, a binding affinity score was predicted and reported as a percentile rank by comparing the peptide’s score against the scores of five million random *n*-mers selected from the UniProt database^23^. (There is no mention in Immune Epitope Database [www.iedb.org] of the source of the 5 million *n*-mers used in generating percentile rank values other than they are selected from the SWISS-PROT database of UniProt^23^). Smaller percentile ranks indicate higher binding affinity. For each gene locus (e.g. DRB1) and spike protein variant (e.g. Delta variant), all alleles and *n*-mers (formatted as a FASTA sequence alignment) were entered as a single query and, thus, the same set of 5 million random *n*-mers was employed to rank all queried alleles.

The IEDB database was queried using the NetMHCIIpan method, which generates scores; higher scores indicate higher affinity. Affinity scores are ranked by the database from high to low, such that the highest score has the lowest percentile rank and the lowest score has the highest percentile rank. Thus, the lower the percentile rank, the higher the binding affinity. In this study, we retrieved and kept the Lowest Percentile Rank (LPR) for each combination of allele and *n*-mer of each spike glycoprotein. Since the success of binding of a specific HLA molecule to a specific peptide critically depends on their affinity, we employed a conservative threshold of LPR < 1 to identify epitope sequences (*n*-mers) with LPR < 1 (highest affinity) for further analysis. We selected this threshold in order to filter out *n*-mers of the viral spike glycoprotein that do not represent high binding affinity of the type that would initiate antibody production. In other words, the lower the affinity the less likely is the recognition of the protein fragment by the HLA molecule and subsequent formation of an effective peptide-HLA molecule complex, necessary to engage CD4 + T lymphocytes in antibody production by B cells. Therefore, analyses based on low affinities are prone to provide the wrong information. By contrast, focusing on high binding affinities (as was done in this study) safeguards against spurious results.

### Vaccine effectiveness (Tables 3 and 4)

**Table 3:** Reported vaccine effectiveness after 2 doses for the SARS-CoV-2 variants indicated in the table. Parentheses are 95% CI (if given in the study).

Wildtype		Alpha		Beta	
Ref	% Effectiveness	Ref	% Effectiveness	Ref	% Effectiveness
^25^	94.1 (89.3–96.8)	^28^	89.5 (85.8–92.3)	^28^	75 (70.5–78.9)
^26^	95 (90.3–97.6)	^29^	100 (91.8–100)	^29^	96.4 (91.9–98.7)
^37^	88 (81–92)	^30^	95.3 (94.9–95.7)	^37^	77 (63–86)
^42^	92 (87–95)	^31^	86.9 (80.4–91.2)	^39^	100 (53.5–100)
^42^	98 (83–100)	^33^	85	^40^	86 (67–94)
**Mean**	**93.4 (86–96)**	^34^	98	^42^	86 (0–98)
		^35^	86 (76–97)	**Mean**	**86.7 (58–93)**
		^36^	89 (79–94)		
		^37^	86 (81–90)		
		^38^	92 (88–95)		
		^38^	89 (87–90)		
		^42^	88 (86–90)		
		^42^	92 (87–95)		
		**Mean**	**90.5 (86–94)**		

Numbers in bold are means of vaccine effectiveness and 95% CI.

**Table 4:** Reported vaccine effectiveness after 2 doses for the SARS-CoV-2 variants indicated in the table. Parentheses are 95% CI (if given in the study).

Gamma		Delta		Omicron	
Ref	% Effectiveness	Ref	% Effectiveness	Ref	% Effectiveness
^42^	**90 (76–96)**	^30^	88 (85.3–90.1)	^27^	65.5 (63.9–67)
		^38^	87 (69–95)	^41^	70 (62–76)
		^43^	75 (71–78)	**Mean**	**67.7 (63–71)**
		^44^	51.9 (47–56.4)		
		^44^	73.1 (67.5–77.8)		
		^45^	82		
		^38^	95 (91–97)		
		^42^	92 (89–94)		
		^42^	94 (90–97)		
		**Mean**	**82.0 (76–86)**		

Numbers in bold are means of vaccine effectiveness and 95% CI.

As a guide for vaccine effectiveness against SARS-CoV-2 variants, the review by Hayawi et al.^2^ was used. Estimates of vaccine effectiveness and associated 95% confidence intervals (when given) of mRNA-based vaccines (BNT162b [Pfizer] and mRNA-1273 [Moderna]) against the Wildtype, Alpha, Beta, Gamma, and Delta variants after 2 doses of vaccine administration were retrieved from the original publications^25–45^.

### Statistical analyses

Standard statistical methods were used to analyze the data, including repeated measures analysis of variance and linear regression. The IBM-SPSS statistical package (version 27) was used for data analysis.

### Monte Carlo analysis

The main objective of this study was to test the hypothesis that the clinical vaccine effectiveness against COVID-19 caused by different variants depends on the level of high affinity binding between peptide (epitope) sequences of the variant-specific spike glycoproteins and 66 common HLA Class II molecules. For that purpose, we used a simple linear regression analysis where the dependent variable was the percent clinical vaccine effectiveness after 2 doses of mRNA COVID-19 vaccines (mean of published studies; Tables 3 and 4) and the independent variable was the derived percent of HLA high affinity bindings determined using an in silico approach. Although the result of this analysis provides the fundamental information about the association between vaccine effectiveness and HLA high affinity binding, we performed an additional analysis using the 95% CI of the vaccine effectiveness estimates to encompass the variability of those estimates in determining the aforementioned association. For that purpose, we used a Monte Carlo approach of repeated random sampling of possible vaccine effectiveness estimates from within their 95% CI, as follows. For each SARS-CoV-2 variant, we used the mean lower and upper 95% CI values (bold in parentheses in Tables 3 and 4) to randomly select a percent vaccine effectiveness estimate lying within that range and regressed it against the in silico derived estimate of HLA high binding affinity for that variant. We carried out that procedure 1000 times and retained the Pearson correlation coefficient, *r*. For statistical purposes, we Fisher z-transformed *r* to normalize its distribution:

1 $${r}_{z}=\mathrm{atanh}(r)$$

Finally, we computed the mean ${r}_{z}$ and its 95% confidence intervals using bias corrected accelerated bootstrap (N = 1000 bootstrap samples, seeded by Mersene Twister) and converted them to *r*:

2 $$r=\mathrm{tanh}({r}_{z})$$

These estimates incorporate the reported variability in vaccine clinical effectiveness in determining its association with HLA high binding affinity.

### Ethical approval

This article does not contain any studies with human participants performed by any of the authors.

## Results

There were 2476 high binding affinity cases with lowest percentile rank LPR < 1 (as discussed above) which included the Wildtype spike glycoprotein. The values and percentages of high affinity binding cases shared between Wildtype and specific VOC are given in Table 5, together with the average vaccine effectiveness of mRNA vaccines.

**Table 5:** HLA high affinity binding and vaccine effectiveness for 6 SARS-CoV-2 variants.

	% HLA high binding affinity	% Vaccine effectiveness
Wildtype	100	93.4
Alpha	95.1	90.5
Beta	98.8	86.7
Gamma	87.6	90.0
Delta	71.6	82.0
Omicron	58.6	67.7

The reported average clinical vaccine effectiveness was strongly and highly significantly associated with the percentage of high affinity binding of VOCs shared with the Wildtype. More specifically, let *E* be the average clinical vaccine effectiveness for a given SARS-CoV-2 variant and *H* the percentage of high HLA binding affinity shared by that variant and the Wildtype; the regression equation of the dependence of *E* on *H* was:

3 $$ E = 41.56 + 0.51H $$

**Figure 2:**
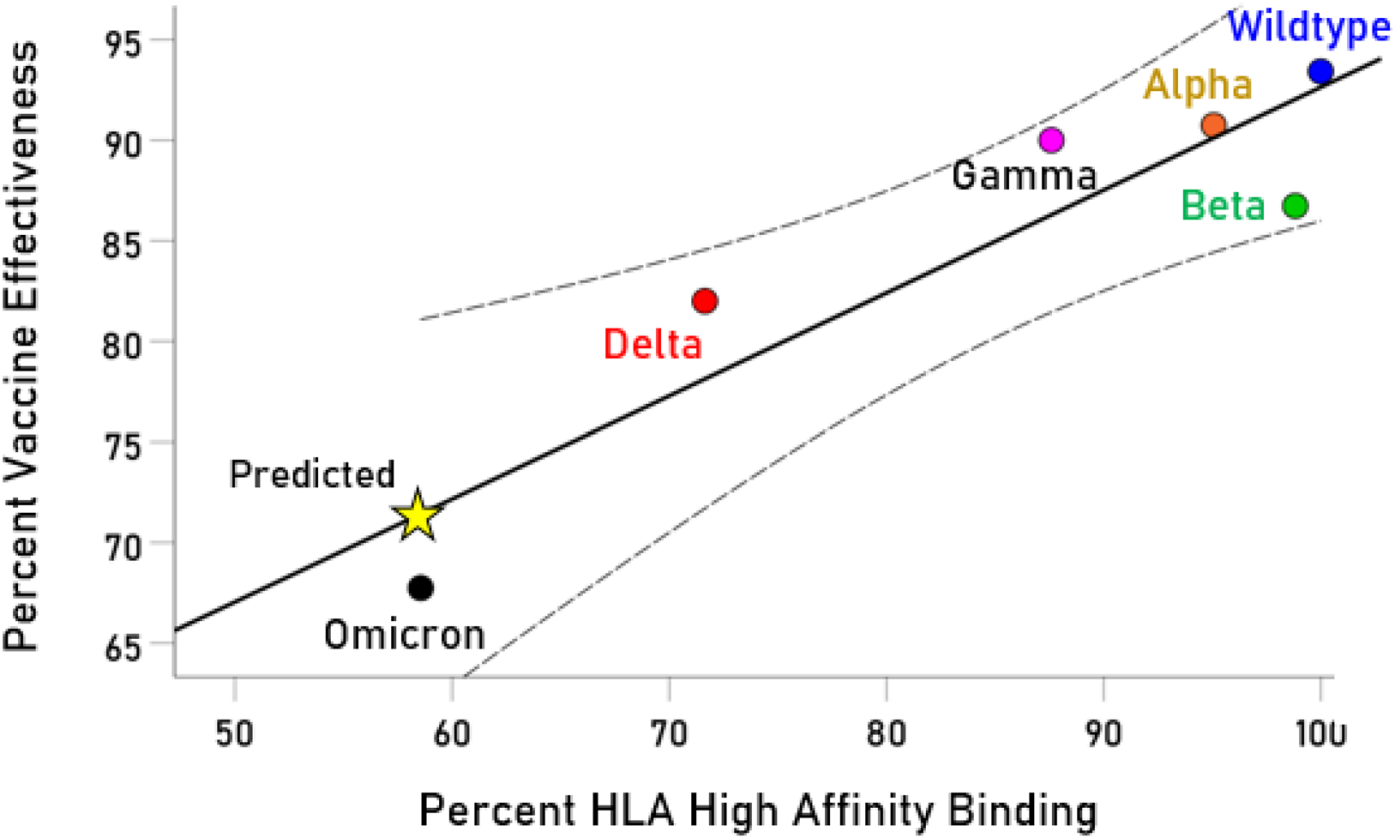
Dependence of vaccine effectiveness on percent of HLA high binding affinity. Curves are 95% mean confidence intervals (CI).

The model was strong (r = 0.910) and statistically highly significant (*P* = 0.013, N = 6). These data are plotted in Fig. 2; it can be seen that all 6 estimates of clinical vaccine effectiveness lie within the 95% CI of the prediction derived from the percent of HLA high binding affinity of these variants. More specifically, the model predicted a 71.4% vaccine effectiveness for Omicron VOC.

The results of the Monte Carlo analysis, incorporating the variability of the vaccine effectiveness estimates, confirmed the robustness of the findings above. The mean correlation and its 95% CI computed from 1000 Monte Carlo samples were 0.827 (0.817, 0.838). Given that the critical value (*P* < 0.05) of the correlation coefficient is 0.707 for N = 6, all three correlation values above are statistically significant at the *P* < 0.05 level. Moreover, the upper 95% CI of 0.838 is significant at the *P* < 0.01 level.

### Other results

**Figure 3:**
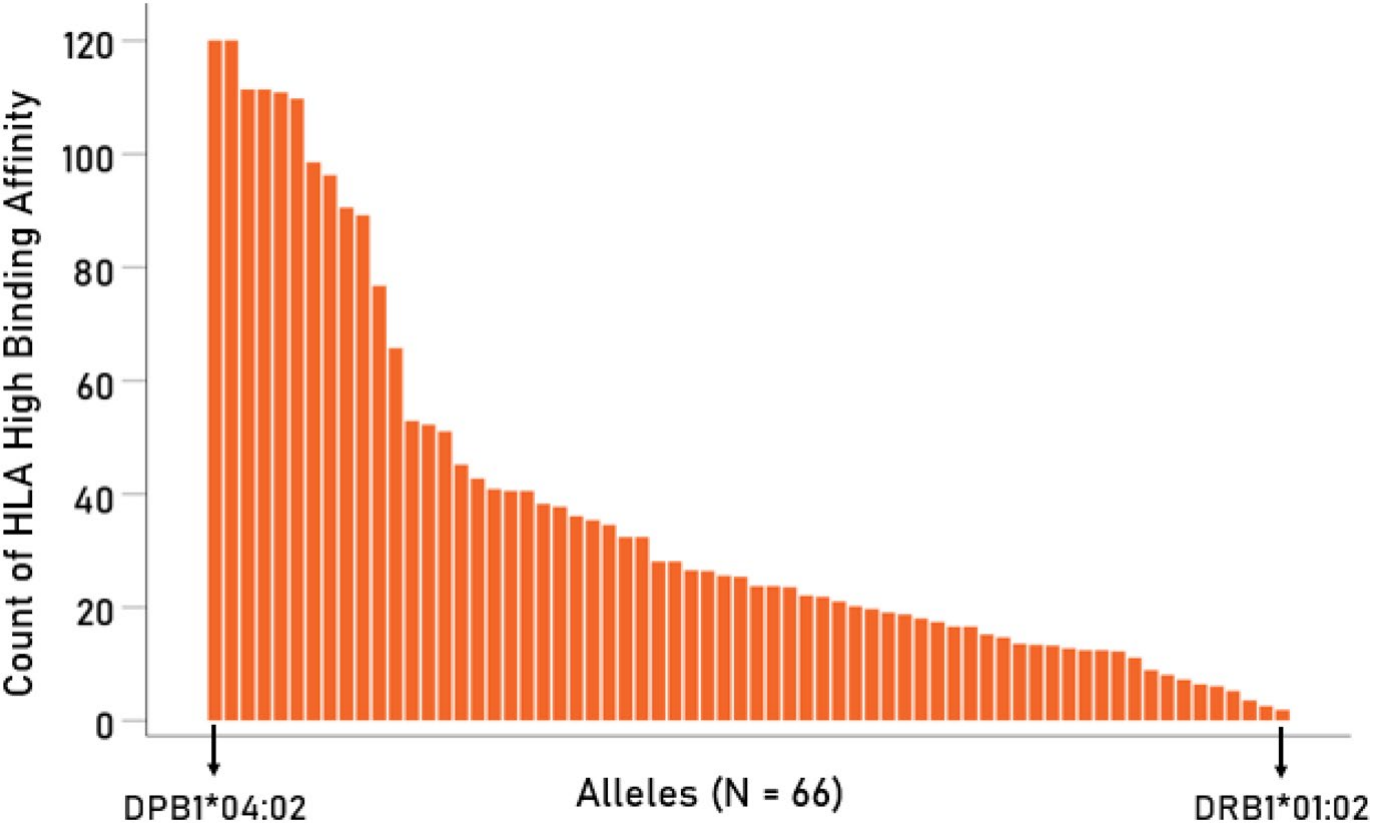
The average counts (per variant) of HLA high binding affinity (LPR < 1) of the 66 alleles used is plotted against their rank, from high to low.

The analyses above were done on 2476 cases (*15/18/22*-mer sequences) for which there was high HLA binding affinity in both the Wildtype and a specific variant. In additional analyses, we assessed the effect of *n*-mer length (15, 18, 22 AA) and HLA gene (DPB1, DQB1, DRB1) on HLA high affinity binding, and also assessed the variability among individual alleles in that respect. Given a HLA binding affinity threshold of LPR < 1, we used the number fulfilling that threshold as the dependent variable in further analyses. We found the following. (a) The *n-*mer length did not have a statistically significant effect on the count of HLA high binding affinity cases (*P* = 0.395, Greenhouse–Geisser test; repeated measures ANOVA where the *n*-mer length was the Within Subjects factor). (b) There was a statistically significant effect of HLA gene (*P* < 0.001, F_[2,63]_  = 14.66, F-test in univariate ANOVA with Gene as a fixed factor); the HLA high binding affinity counts for the DPB1 gene were significantly higher than those for either DQB1 or DRB1 genes (*P* < 0.001 for each), whereas there was no significant difference between DQB1 and DRB1 (*P* = 0.362). Finally, (c) there was a substantial variation in the HLA high binding affinity counts among the 66 alleles tested (Fig. 3), with allele DPB1*04:02 having ~ 60 × higher counts than DRB1*01:02 (Fig. 3). The trend depicted in Fig. 3 was similar across the 6 spike glycoproteins and 3 *n-*mer lengths.

## Discussion

Here we document that SARS-CoV-2 vaccine effectiveness for each VOC is highly related to the percent of HLA Class II binding affinity shared with the Wildtype strain against which the vaccines were developed. That is, as VOC have evolved they share progressively less in common with the Wildtype strain with regard to HLA Class II high-affinity binding. Given the role of HLA in facilitating antibody production in the event of future exposure, the very goal of vaccination, these findings of reduced high-affinity binding may partially account for some of the reductions in vaccine effectiveness that have been reported with successive VOC^2^. The lower vaccine effectiveness against Omicron^27,41^ is consistent with mounting literature documenting enhanced immune evasion with Omicron^45–47^. Finally, the predictive estimate of 71.4% of vaccine effectiveness against Omicron is close to the 65.5% vaccine effectiveness reported recently^27^.

Our study points to a mechanism that partially underlies the reduced vaccine effectiveness against Omicron, namely, reduced likelihood that exposure to Omicron will induce an antibody response due to relatively low overlap of high-affinity HLA binding with the vaccine-associated Wildtype strain. It should be noted that, although reduced relative to other VOC, Omicron shares 58.6% high-affinity HLA binding capability with the Wildtype strain, suggesting that a considerable degree of immune protection may be conferred albeit less than with prior VOC. However, it should be pointed out that our approach of predicting vaccine effectiveness extends to all currently identified SARS-CoV-2 VOCs (Fig. 2).

In light of findings pointing to reduced effectiveness of vaccines as SARS-CoV-2 evolves^2^ and the unprecedented spread of Omicron, it becomes increasingly imperative that vaccines and therapeutics (e.g., monoclonal/polyclonal antibodies) also evolve to curb the pandemic and reduce the likelihood of new VOCs developing. Although prior to identification of the Omicron variant, we documented 18 epitopes that are shared across other VOCs and bind with high affinity to HLA Class II alleles^22^, paving the way for future studies evaluating the suitability of those epitopes for SARS-CoV-2 vaccine development. The present findings documenting a strong correspondence between in silico HLA binding affinity with VOC and clinical data on vaccine effectiveness highlight the utility of HLA-epitope in silico studies in predicting future vaccine effectiveness for new SARS-CoV-2 variants. It should also be noted that our approach extends beyond the current vaccines based on the Wildtype. For example, if a new vaccine is developed specifically against the Omicron spike glycoprotein, its effectiveness for other variants could be predicted based on in silico determination of HLA high binding affinity of sequences shared by the Omicron spike glycoprotein and those of other variants. Furthermore, similar considerations can be advanced for other vaccines, in general, where multiple strains are involved, such as influenza and pneumococcus.

Although the present findings point to an intriguing link between SARS-CoV-2 vaccine effectiveness and HLA binding affinity, the following study qualifications must be considered. First, vaccine effectiveness was based on completion of two vaccine doses and does not preclude potential benefit from a booster doses as has been recently documented^10,48^. Second, only data from clinical trials using mRNA-based vaccines were analyzed for reasons of data homogeneity, since different results have been reported for different kinds of vaccines. And third, we evaluated the binding affinity SARS-CoV-2 variants with a small subset of HLA Class II alleles, albeit the most common alleles. However, the strong results obtained in predicting vaccine effectiveness underscore the utility of that set. Finally, it should be noted that this study was not aimed at, nor addressed issues on, designing new, more effective vaccines^49^ or investigating the various mechanism s affecting (enhancing or reducing) vaccine effectiveness^50,51^. However, our results are relevant to the finding^52^ that “mRNA-based vaccination of humans induces a persistent germinal centre B cell response, which enables the generation of robust humoral immunity” (abstract^52^). This finding was obtained in a sample of individuals who had received 2 doses of BNT162b2, a mRNA-based vaccine. Indeed, based on the results of our study, we hypothesize that these individuals carried HLA Class II alleles with high binding affinity to the Wildtype spike glycoprotein, since the successful formation of a glycoprotein segment—HLA Class II molecule complex is a prerequisite of B cell activation and subsequent antibody production^3^.

## Study limitations

This study was performed exclusively in silico and thus the data presented are subject to any constraints that may affect HLA binding affinity predictive tools (NetMHCIIpan in this case). Although important to consider, this limitation is mitigated in part by the fact that all predictive methods of the IEDB suite are trained using experimental datasets. In spite of this limitation, we believe that computational studies can offer important insights concerning binding affinity prediction and help save valuable time when combating a global public health crisis. Furthermore, this study did not incorporate the effect of post-translational modifications, such as glycosylation, on the spike glycoprotein and their effects on HLA binding. Glycosylation can have a major influence on protein antigen uptake and proteolytic processing, which in turn can affect MHC presentation and subsequent immune responses^51^. Our main interest was to perform a comprehensive sequence-level analysis of the binding affinity of the spike glycoprotein for the 6 major VOC of the SARS-CoV-2 virus.

## Supplementary Information

Supplementary Information.

## Data Availability

Sources and accessions for all glycoprotein sequences are given in the Appendix.
